# Dipstick proteinuria predicts all-cause mortality in general population: A study of 17 million Korean adults

**DOI:** 10.1371/journal.pone.0199913

**Published:** 2018-06-28

**Authors:** Yeongkeun Kwon, Kyungdo Han, Yang Hyun Kim, Sungsoo Park, Do Hoon Kim, Yong Kyun Roh, Yong-Gyu Park, Kyung-Hwan Cho

**Affiliations:** 1 Department of Family Medicine, Korea University College of Medicine, Seoul, Republic of Korea; 2 Center for Obesity and Metabolic Diseases, Korea University Anam Hospital, Seoul, Republic of Korea; 3 Department of Biostatistics, College of Medicine, The Catholic University of Korea, Seoul, Republic of Korea; 4 Division of Upper Gastrointestinal Surgery, Department of Surgery, Korea University College of Medicine, Seoul, Republic of Korea; 5 Department of Family Medicine, Hallym University College of Medicine, Chuncheon, Republic of Korea; The University of Tokyo, JAPAN

## Abstract

**Objective:**

A quantitative basis for the use of dipstick urinalysis for risk assessment of all-cause mortality is scarce. Therefore, we investigated the association between dipstick proteinuria and all-cause mortality in a general population and evaluated the effect of confounders on this association.

**Methods:**

The study population included 17,342,956 adults who underwent health examinations between 2005 and 2008 under the National Health Insurance System. Proteinuria was determined using a single dipstick urinalysis, and the primary outcome of this study was all-cause mortality. The prognostic impact of proteinuria was assessed by constructing a multivariable Cox model.

**Results:**

The mean age of the study population (53.24% male) was 46.06 years; 724,681 deaths from all causes occurred over a median follow-up period of 9.34 years (interquartile range 8.17–10.16), and the maximum follow-up was 12.12 years. After full adjustment for covariates, a higher level of dipstick proteinuria indicated a higher risk of all-cause death [Hazard ratios (95% confidence intervals); 1.22 (1.20–1.24), 1.47 (1.45–1.49), 1.81 (1.77–1.84), 2.32 (2.24–2.41), 2.74 (2.54–2.96); trace to 4+, respectively], and various subgroup analyses did not affect the main outcome for the total population. ≥1+ proteinuria in the group without metabolic diseases (hypertension, diabetes, dyslipidemia, or obesity) resulted in higher hazard ratios than those in the group with metabolic diseases and negative or trace proteinuria.

**Conclusions:**

Our study showed a strong association between dipstick proteinuria and all-cause mortality in this nationwide population-based cohort in South Korea.

## Introduction

Proteinuria is a key feature of chronic kidney disease (CKD),[1,2] and has close association with increased mortality.[3–5] Although population surveys demonstrated the occurrence of excessive proteinuria among individuals with hypertension and diabetes,[6,7] proteinuria is associated with increased mortality independent of the presence of hypertension or diabetes.[3–5] Despite its role in negative health outcomes, proteinuria is usually asymptomatic, and public awareness on this condition is low,[6,8] with > 90% of CKD patients being unaware of their condition.[9,10] Therefore, availability of simpler screening tests would improve detection of proteinuria in clinical settings. It is important to study the correlation between the results of screening tests and the risk of mortality to improve public awareness in an effort to prevent CKD-induced complications.

Current guidelines pertaining to the detection of proteinuria may not be sufficient to obtain and accurately reflect data regarding the association between proteinuria and mortality risk in the general population. Guidelines recommend the urine protein-to-creatinine ratio (PCR) or the urine albumin-to-creatinine ratio (ACR) as the preferred measure of proteinuria.[11–13] However, PCR or ACR is not considered practical for large epidemiological studies because they are slow to produce results, are expensive, and are inconvenient to perform. Moreover, recommendations support opportunistic targeted screening in primary-care settings.[11,12,14] However, high-risk populations such as elderly patients or those with hypertension or diabetes would not represent the general population and this could cause a selection bias when assessing the mortality risk in the general population.

Dipstick urinalysis could be useful in assessing the association between proteinuria and mortality in the general population owing to several advantages: 1) A dipstick urinalysis test is inexpensive, widely available, easy to perform, and provides a rapid result at the point of care. These factors favor its application in large-scale epidemiological studies and for screening in non-risk populations in whom screening for proteinuria is not recommended by guidelines. 2) Dipstick test result ≥ trace identifies ACR ≥30mg/g with 43–69% sensitivity and 86–93% specificity.[15,16] Because the risk of false negative cases is lower, despite its relatively limited use, it can be inferred that the hazard ratio (HR) calculated based on dipstick urinalysis would be an estimation of the minimal mortality risk.[15,16]

A nationwide health screening program is available in South Korea to approximately 98% of the overall population that has subscribed to the National Health Insurance (NHI).[17] Evaluation of the claims data of the NHI in South Korea shows data obtained from a homogeneous population indicating that these data can be generalized to the Korean population. We obtained results of dipstick urinalysis in approximately 17 million adults who had been medically screened using a standardized process and followed up on their vital status since 2005. We aimed to investigate the association between dipstick proteinuria and all-cause mortality by controlling the effect of confounders on this association.

## Materials and methods

### Data source and study population

The Health Insurance Review and Assessment (HIRA) database included in the NHI system contains data on health insurance claims of approximately 98.0% of South Koreans.[18,19] Among the 31,237,363 adults (age ≥ 20 years) who underwent health examinations between 2005 and 2008, we excluded 117,440 subjects with insufficient data, 13,776,911 subjects in whom more than one health examination had been performed, and 56 subjects who were reported dead prior to the health examination owing to an administrative error. Therefore, our study population comprised 17,342,956 subjects. The year of the first health examination was considered the index year. This population was monitored from the start of the index year to the date of death or until December 31, 2015, whichever was earlier. Our study was approved by the Institutional Review Board of Korea National Institute for Bioethics Policy (No. P01–201603–21–005). Because information pertaining to the subjects was anonymized and de-identified prior to the analyses, the requirement of informed consent was waived for this study.

### Dipstick urinalysis and main outcome measures

Proteinuria was determined by a single dipstick urinalysis. Urine samples were obtained early in the morning following an overnight fast, and the results of the dipstick urinalysis were interpreted on the basis of a color scale that semi-quantified proteinuria as negative, trace, 1+, 2+, 3+, or 4+. The primary outcome of this study was all-cause mortality, which is a robust and unbiased index that does not require an adjudication to avoid clinical assessments or documentation of biases.[20] The cause of death was identified on the basis of the Tenth Revision of the International Classification of Disease (ICD-10).

### Covariates

Diabetes was defined on the basis of the following criteria: (1) at least one claim in a year for a prescription of antidiabetic medications (ICD-10 codes E11–14) or (2) a fasting plasma glucose level ≥126 mg/dL (obtained from the health examination database). Hypertension was defined on the basis of at least one claim in a year for a prescription of antihypertensive medication (ICD-10 codes I10–I15) or systolic/diastolic blood pressure ≥140/90 mmHg when measured on two or more separate occasions. Dyslipidemia was defined by at least one claim in a year for a prescription of antidyslipidemic medications (ICD-10 code E78) or a total serum cholesterol level ≥240 mg/dL (obtained from the health examination database).

Body-mass index (BMI) was calculated as the weight in kilograms divided by the square of the height in meters. A BMI of 25 kg/m^2^ was used as the cut-off value to define obesity for the Asian population enrolled in our study.[21] Participants were categorized as non-smokers, ex-smokers, or current smokers, and the frequency of alcohol consumption was categorized as 0, 1–2, or ≥3 times/week on the basis of data obtained from the questionnaire. Regular exercise indicated strenuous physical activity for at least 20 min/day, and the frequency of exercise was categorized as 0, 1–4, or ≥5 times/week. Hospitals certified by the NHI system performed these health examinations and conducted regular quality control surveys.

### Statistical analyses

Data were expressed as means (standard deviation [SD]), geometric means (95% confidence interval [CI]), or percentages. Continuous variables with non-normal distribution are presented as medians and interquartile ranges (IQR). Using a multivariable Cox proportional hazard model and the negative proteinuria group as a reference, hazard ratios (HRs) and 95% CIs for all-cause mortality observed in the six study groups were analyzed on the basis results of dipstick urinalysis. Proportional hazard assumptions were evaluated using the logarithm of the cumulative hazards function and Kaplan-Meier estimates for each group. Variation in the effect of proteinuria observed between subgroups was determined by calculating the HRs for mortality on the basis of covariates, and we tested the interaction between group assignments and risk factor categories. Furthermore, the prognostic impact of proteinuria based on weight of subjects was assessed by constructing a multivariable Cox model for the four subgroups with categories containing two variables (proteinuria [negative/trace vs. ≥1+], and BMI [< 25 kg/m^2^ vs. ≥25 kg/m^2^]). Similarly, the prognostic impact of proteinuria was assessed on the basis of the presence of metabolic diseases (hypertension, diabetes, or dyslipidemia) by constructing a multivariate Cox model for the four subgroups, with categories containing two variables (proteinuria [negative/trace vs. ≥1+], and metabolic diseases [none vs. at least one]). Statistical analyses were performed using the SAS software version 9.3 (SAS Institute Inc., Cary, NC, USA). A two-tailed *p*-value of <0.05 was considered statistically significant.

## Results

### Baseline characteristics

The mean age of the study population was 46.06 years (SD 14.59 years), among which 53.24% were men (Table 1). Mean BMI was 21.90 kg/m^2^ (SD 1.99 kg/m^2^) in the non-obese and 27.24 kg/m^2^ (SD 2.38 kg/m^2^) in the obese group. Participants with a smoking history (including ex- and current smokers) comprised 33.13% of the total population. We observed that 52.75% of the population reported no alcohol consumption and 54.49% reported they did not exercise at all. Participants diagnosed with hypertension, diabetes, and dyslipidemia comprised 26.31%, 7.90%, and 14.36% of the total population at baseline, respectively. Overall, we observed that participants with a higher level of dipstick proteinuria were more likely to be older and have a diagnosis of hypertension, diabetes, and dyslipidemia.

**Table 1:** Baseline characteristics of the study population.

		Dipstick proteinuria categories					
		Negative	Trace	1+	2+	3+	4+
Participants, No. (%)		16,766,456 (96.68)	264,561 (1.53)	209,593 (1.21)	81,781 (0.47)	17,347 (0.10)	3,218 (0.02)
Age, No. (%)	20–29	2,734,135 (16.31)	36,964 (13.97)	23,853 (11.38)	7,666 (9.37)	1,374 (7.92)	257 (7.99)
	30–39	3,245,003 (19.35)	43,787 (16.55)	27,578 (13.16)	9,116 (11.15)	1,748 (10.08)	312 (9.7)
	40–49	4,153,203 (24.77)	66,649 (25.19)	48,472 (23.13)	17,922 (21.91)	3,678 (21.2)	688 (21.38)
	50–59	3,316,059 (19.78)	55,848 (21.11)	47,622 (22.72)	19,598 (23.96)	4,276 (24.65)	773 (24.02)
	60–69	2,138,950 (12.76)	37,920 (14.33)	37,470 (17.88)	16,254 (19.88)	3,757 (21.66)	696 (21.63)
	70–79	1,016,522 (6.06)	19,701 (7.45)	20,668 (9.86)	9,464 (11.57)	2,113 (12.18)	419 (13.02)
	≥ 80	162,584 (0.97)	3,692 (1.4)	3,930 (1.88)	1,761 (2.15)	401 (2.31)	73 (2.27)
Men, No. (%)		8,923,035 (53.22)	141,626 (53.53)	111,417 (53.16)	45,172 (55.24)	9,882 (56.97)	1,884 (58.55)
BMI, mean (SD), kg/m^2^		23.57 (3.25)	23.91 (3.41)	24.24 (3.57)	24.47 (3.72)	24.52 (3.74)	24.50 (3.79)
Hypertension, No. (%)		4,317,065 (25.75)	91,917 (34.74)	95,904 (45.76)	45,779 (55.98)	10,975 (63.27)	2,089 (64.92)
Diabetes, No. (%)		1,256,884 (7.5)	36,358 (13.74)	45,480 (21.7)	23,827 (29.14)	6,438 (37.11)	1,279 (39.75)
Dyslipidemia, No. (%)		2,358,503 (14.07)	50,298 (19.01)	49,802 (23.76)	24,260 (29.66)	6,421 (37.02)	1,300 (40.4)
Smoking, No. (%)	Non	11,210,000 (66.86)	175,504 (66.34)	141,726 (67.62)	54,914 (67.15)	11,689 (67.38)	2,120 (65.88)
	Ex	1,399,876 (8.35)	24,133 (9.12)	18,872 (9)	7,902 (9.66)	1,701 (9.81)	328 (10.19)
	Current	4,155,807 (24.79)	64,924 (24.54)	48,995 (23.38)	18,965 (23.19)	3,957 (22.81)	770 (23.93)
Alcohol consumption, No. (%)	No	8,834,661 (52.69)	139,510 (52.73)	116,247 (55.46)	46,408 (56.75)	10,352 (59.68)	1,911 (59.38)
	1–2 per week	6,414,233 (38.26)	97,712 (36.93)	69,892 (33.35)	25,990 (31.78)	5,027 (28.98)	967 (30.05)
	≥3 per week	1,517,562 (9.05)	27,339 (10.33)	23,454 (11.19)	9,383 (11.47)	1,968 (11.34)	340 (10.57)
Exercise, No. (%)	None	9,139,221 (54.51)	140,139 (52.97)	114,800 (54.77)	45,005 (55.03)	9,531 (54.94)	1,754 (54.51)
	1–4 per week	6,273,644 (37.42)	100,131 (37.85)	74,125 (35.37)	28,160 (34.43)	5,929 (34.18)	1,110 (34.49)
	≥ 5 per week	1,353,591 (8.07)	24,291 (9.18)	20,668 (9.86)	8,616 (10.54)	1,887 (10.88)	354 (11)

Abbreviations: BMI: body mass index. SD: standard deviation

Proteinuria was determined by a single dipstick urinalysis. Urine samples were obtained early in the morning following an overnight fast, and the results of the dipstick urinalysis were interpreted on the basis of a color scale that semi-quantified proteinuria as negative, trace, 1+, 2+, 3+, or 4+.

BMI is calculated as weight in kilograms divided by height in meters squared.

### All-cause mortality

We observed 724,681 deaths from all causes to have occurred over a median follow-up period of 9.34 years (interquartile range 8.17–10.16), and the maximum follow-up duration was 12.12 years. The crude incidence rates of all-cause mortality per 1000 person-years by dipstick proteinuria categories are shown in Table 2. A higher level of dipstick proteinuria might indicate a higher incidence of all-cause death.

**Table 2:** Association between dipstick proteinuria and all-cause mortality.

		Event	Duration	Incidence rate (per 1000 person-years)	Hazard ratios (95% confidence intervals)			
					Model 1	Model 2	Model 3	Model 4
Dipstick urinalysis	Negative	675,900	119,492,876.6	5.6564	1.000	1.000	1.000	1.000
	Trace	15,376	1,854,585.97	8.2908	1.447 (1.424–1.470)	1.255 (1.235–1.275)	1.285 (1.264–1.305)	1.216 (1.196–1.235)
	1+	19,123	1,459,719.87	13.1005	2.174 (2.143–2.205)	1.578 (1.556–1.601)	1.668 (1.644–1.692)	1.467 (1.446–1.489)
	2+	10,563	560,562.41	18.8436	2.951 (2.895–3.009)	2.015 (1.976–2.054)	2.196 (2.154–2.238)	1.807 (1.773–1.843)
	3+	3,067	116,199.45	26.3943	3.983 (3.844–4.127)	2.643 (2.551–2.739)	2.946 (2.844–3.053)	2.322 (2.241–2.406)
	4+	652	21,074.66	30.9376	4.674 (4.329–5.047)	3.275 (3.033–3.536)	3.493 (3.235–3.772)	2.738 (2.536–2.957)

Abbreviations: BMI: body mass index

Model 1 is not adjusted for any covariates.

Model 2 is adjusted for age and sex.

Model 3 is additionally adjusted for BMI, alcohol consumption, exercise, and smoking status.

Model 4 is adjusted for metabolic diseases including hypertension, diabetes, and dyslipidemia in addition to Model 3 variables.

The correlation between all-cause mortality and dipstick proteinuria was determined by constructing four Cox regression models with gradual adjustments of covariates. After full adjustments for age, sex, BMI, lifestyle variables (alcohol consumption, exercise, and smoking), and metabolic diseases (hypertension, diabetes, and dyslipidemia) in model 4, trace proteinuria was observed to be associated with a ≥20% increase in the mortality risk (HR 1.21, 95% CI 1.20–1.24). Additionally, 3+ and 4+ proteinuria was observed to be associated with a 2-fold or higher mortality risk than that of negative proteinuria (HR 2.32, 95% CI, 2.24–2.41, and HR 2.74 95% CI 2.54–2.96, respectively). Adjustment for covariates weakened the association between the levels of proteinuria and mortality risk, but maintained the significance of the mortality risk.

### Subgroup analyses

The association between dipstick proteinuria and all-cause mortality remained significant in all subgroups using a few covariates; however, a higher risk of all-cause mortality was observed in men and in subgroups with metabolic diseases (hypertension, diabetes, or dyslipidemia) (Table 3). Participants aged <65 years showed higher HRs than participants aged ≥65 years, except with regard to trace proteinuria. Participants belonging to the obese subgroup demonstrated a higher risk of all-cause mortality than those belonging to the non-obese subgroup, except with regard to 4+ proteinuria. No significant interaction was identified between the study groups. A comparison of proteinuria levels (negative and trace vs. ≥1+) and weight (BMI <25 kg/m^2^ vs. ≥25 kg/m^2^) between the four subgroups indicated that the subgroup comprising participants without obesity and with ≥1+ proteinuria presented the highest risk of all-cause mortality (HR 1.66, 95% CI 1.64–1.68) compared with the two subgroups comprising participants without obesity (Table 4A). In the fully adjusted model 4, the HR indicated a weaker association between the four subgroups than that in crude model 1; however, the correlation remained significant.

**Table 3:** All-cause mortality according to various covariates and dipstick proteinuria levels.

	Dipstick urinalysis	Hazard ratios (95% confidence intervals)						
		Age	Sex	Diabetes	Hypertension	Dyslipidemia	Metabolic diseases	Weight status
Subgroup 1	Negative	1.000	1.000	1.000	1.000	1.000	1.000	1.000
	Trace	1.190 (1.159–1.222)	1.184 (1.161–1.208)	1.183 (1.160–1.206)	1.184 (1.153–1.216)	1.205 (1.183–1.228)	1.167 (1.128–1.206)	1.217 (1.194–1.241)
	1+	1.571 (1.534–1.610)	1.483 (1.457–1.510)	1.438 (1.411–1.466)	1.438 (1.399–1.479)	1.480 (1.455–1.506)	1.419 (1.368–1.471)	1.510 (1.484–1.537)
	2+	2.096 (2.030–2.163)	1.854 (1.811–1.899)	1.795 (1.747–1.846)	1.772 (1.700–1.848)	1.818 (1.775–1.861)	1.716 (1.619–1.820)	1.911 (1.866–1.957)
	3+	3.014 (2.853–3.184)	2.351 (2.251–2.455)	2.247 (2.125–2.377)	2.245 (2.063–2.443)	2.282 (2.179–2.390)	2.106 (1.854–2.392)	2.435 (2.330–2.544)
	4+	3.519 (3.131–3.955)	2.827 (2.580–3.099)	2.402 (2.111–2.733)	2.617 (2.154–3.181)	2.782 (2.513–3.081)	1.981 (1.422–2.758)	2.691 (2.445–2.961)
Subgroup 2	Negative	1.000	1.000	1.000	1.000	1.000	1.000	1.000
	Trace	1.216 (1.192–1.241)	1.259 (1.226–1.293)	1.274 (1.240–1.310)	1.226 (1.202–1.251)	1.22 (1.181–1.259)	1.225 (1.203–1.248)	1.181 (1.148–1.215)
	1+	1.450 (1.424–1.476)	1.518 (1.482–1.556)	1.585 (1.550–1.620)	1.521 (1.496–1.547)	1.522 (1.481–1.564)	1.513 (1.489–1.537)	1.429 (1.393–1.465)
	2+	1.780 (1.737–1.824)	1.965 (1.900–2.032)	2.004 (1.950–2.059)	1.932 (1.890–1.975)	2.028 (1.961–2.097)	1.917 (1.879–1.957)	1.783 (1.725–1.843)
	3+	2.133 (2.036–2.235)	2.638 (2.479–2.807)	2.599 (2.482–2.722)	2.498 (2.402–2.598)	2.682 (2.536–2.836)	2.478 (2.388–2.571)	2.332 (2.195–2.478)
	4+	2.490 (2.249–2.757)	2.917 (2.536–3.355)	3.186 (2.895–3.506)	2.934 (2.699–3.189)	2.934 (2.611–3.297)	2.941 (2.717–3.182)	3.018 (2.654–3.431)

Subgroup 1 comprises subgroups comprising patients <65 years of age, women, those with body mass index <25 kg/m^2^, and without metabolic diseases (hypertension, diabetes, or dyslipidemia). Subgroup 2 comprises the opposite subgroups.

**Table 4:** Prognostic impact of dipstick proteinuria with respect to weight and metabolic diseases.

**(A)**	Hazard ratios (95% confidence intervals)			
	Non-obese		Obese	
	Negative or trace	≥ 1+	Negative or trace	≥ 1+
Model 1	1.000	2.668 (2.632–2.704)	0.858 (0.853–0.862)	1.971 (1.935–2.009)
Model 2	1.000	1.848 (1.824–1.874)	0.820 (0.816–0.824)	1.415 (1.389–1.441)
Model 3	1.000	1.860 (1.835–1.885)	0.830 (0.825–0.834)	1.433 (1.406–1.460)
Model 4	1.000	1.657 (1.635–1.680)	0.800 (0.796–0.805)	1.238 (1.215–1.262)
**(B)**	Hazard ratios (95% confidence intervals)			
	No metabolic diseases		≥ 1 metabolic diseases (hypertension, diabetes, or dyslipidemia)	
	Negative or trace	≥ 1+	Negative or trace	≥1+
Model 1	1.000	1.709 (1.659–1.761)	3.483 (3.466–3.500)	7.448 (7.357–7.539)
Model 2	1.000	1.518 (1.473–1.564)	1.204 (1.198–1.210)	2.185 (2.158–2.211)
Model 3	1.000	1.513 (1.469–1.559)	1.217 (1.211–1.223)	2.216 (2.189–2.244)
Model 4	1.000	1.513 (1.468–1.559)	1.352 (1.345–1.359)	2.521 (2.490–2.553)

Non-obese is defined as a body mass index (BMI) of 18.5–24.9 kg/m^2^, obese is defined as a BMI of ≥25 kg/m^2^.

Refer to Table 2 regarding the descriptions of model construction.

**Fig 1:**
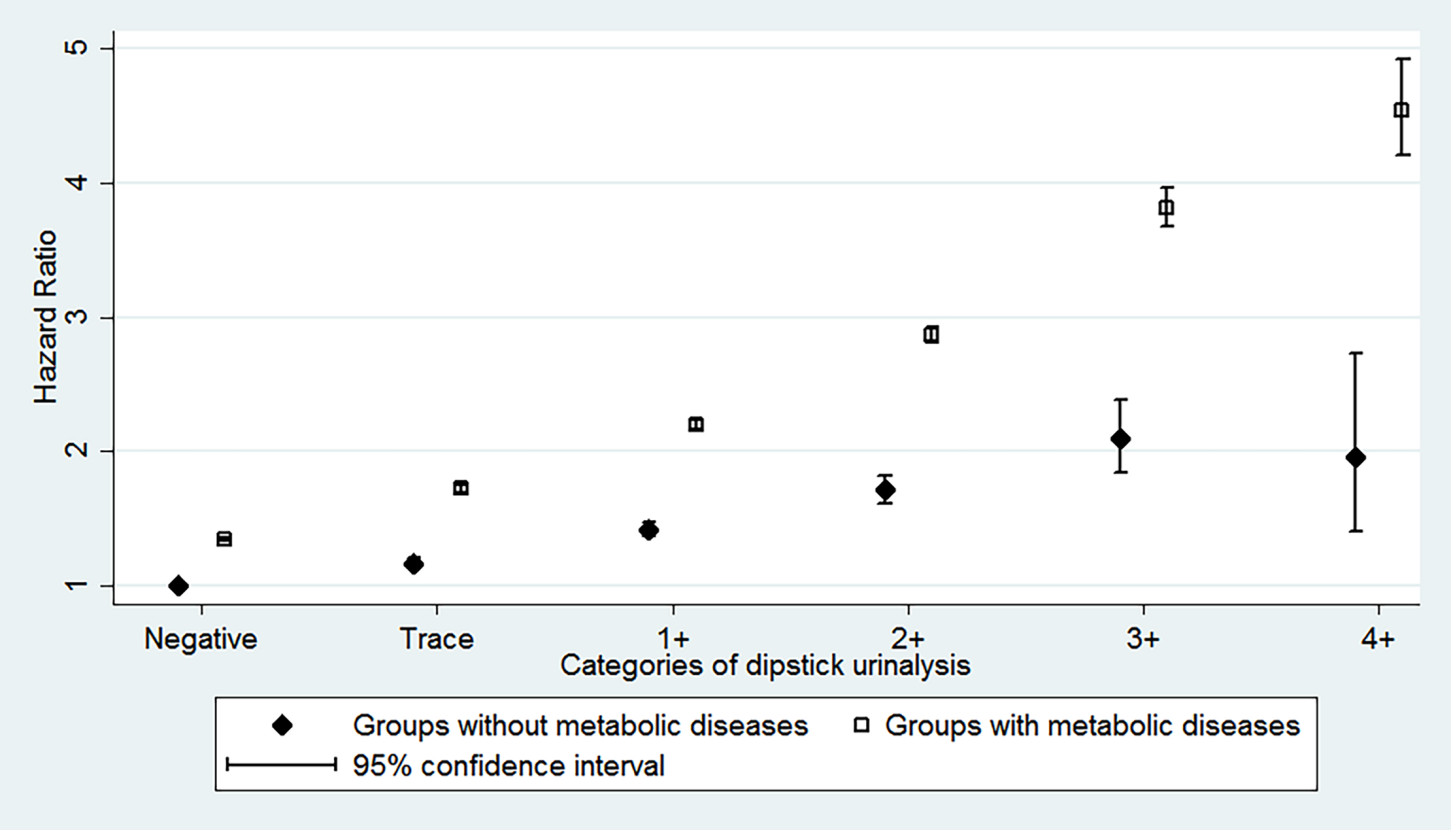
Distribution of hazard ratios of subgroups based on dipstick proteinuria categories and presence of metabolic diseases. The Cox regression model is adjusted for age, sex, body mass index, smoking, alcohol consumption, and exercise. Hazard ratios are calculated using the subgroup without proteinuria and metabolic diseases (hypertension, diabetes, or dyslipidemia) as a reference. Closed diamonds represent hazard ratios of subgroups without metabolic diseases, and open squares represent hazard ratios of subgroups with at least one metabolic disease. Error bars display 95% confidence intervals.

A comparison of the proteinuria levels (negative and trace vs. ≥1+) and the presence of metabolic diseases (yes vs. no, where yes indicated ≥1 metabolic diseases, including hypertension, diabetes, or dyslipidemia) between the four subgroups indicated that the risk of all-cause mortality in the subgroup with metabolic diseases and ≥1+ proteinuria increased by >2-fold (HR 2.52, 95% CI 2.49–2.55) compared to that in the reference group (negative or trace proteinuria without metabolic diseases) (Table 4B). In the fully adjusted model 4, ≥1+ proteinuria in the group without metabolic diseases showed a higher HR than that in the group with metabolic diseases and negative or trace proteinuria (HR 1.51, 95% CI 1.47–1.56, and HR 1.35, 95% CI 1.35–1.36, respectively). Even after adjustment for covariates, the association maintained significance, although the significance was lower than that in the crude model. Fig 1 presents the HRs of the subgroups based on metabolic diseases (yes vs. no, where yes indicates presence of ≥1 metabolic diseases including hypertension, diabetes, or dyslipidemia) across different levels of proteinuria. For all proteinuria categories, the association in the subgroups with metabolic diseases was stronger than that in the subgroups without metabolic diseases. However, the subgroup without metabolic diseases with ≥1+ proteinuria showed a higher risk of all-cause mortality than the subgroup with metabolic diseases but without proteinuria.

## Discussion

To our knowledge, this is the largest population-based study from East Asia to assess the association between dipstick proteinuria and all-cause mortality. We used nationwide and long-term follow-up data to show that dipstick proteinuria was associated with all-cause mortality independent of possible confounders in Korean adults. The consistent results observed with respect to the main outcome in the covariate-adjusted analysis and the subgroup analysis indicate that dipstick proteinuria may be a useful direct marker of mortality, and these results corroborate the robustness of our findings.

The HRs observed in the current study should be interpreted carefully. When used for the detection of proteinuria in the general population, dipstick urinalysis demonstrates a high NPV. A study comprising 10,944 Australian adults showed that a negative dipstick result (<trace) demonstrated an NPV of 97.6% (95% CI 97.2–97.9) for ACR ≥ 30 mg/g and an NPV of 100% (99% CI 99.9–100) for ACR ≥ 300 mg/g.[16] A study comprising 20,759 Korean adults showed that a dipstick reading <trace demonstrated an NPV of 95.5% for ACR ≥ 30 mg/g and a dipstick reading <1+ demonstrated an NPV of 99.8% for ACR ≥ 300 mg/g.[15] Because of the high NPV of dipstick urinalysis when used for the general population, the probability of false negative cases is low/minimal. Therefore, the HRs presented in this study could be interpreted as an estimation of the minimal mortality risk. Because of the limitation of a high false-positive rate associated with a dipstick proteinuria in the general population,[15,16] several previous studies have defined persistent proteinuria as ≥2 times the dipstick positive value. However, this method was seen to lead to a decreased/lower NPV;[22] thus, we used a single dipstick urinalysis measurement to minimize the incidence of false-negative cases.

Considering that disease prevalence can be a significant determinant of efficacy of the dipstick urinalysis test,[23,24] the variability of the association between single dipstick proteinuria and mortality could be explained by differences in the study population. The first National Health and Nutrition Examination Survey reported that proteinuria in trace levels or higher is associated with 71% higher total mortality than that in the absence of proteinuria in men.[25] The Framingham Study reported that dipstick analysis showing trace levels of or ≥1+ proteinuria were associated with a 30–40% increase in the 17-year total mortality.[26] Dipstick proteinuria was associated with a 43% increase in mortality in Italy.[27] In our study, trace and 1+ proteinuria was associated with a 21% and 46% higher all-cause mortality rate, respectively, compared to the no-proteinuria population.

It has been suggested that excess body weight is associated with glomerular hemodynamic changes, primarily increased renal plasma flow, and glomerular hyperfiltration, which predispose patients with obesity to proteinuria.[28,29] Therefore, the confounding effect of obesity should be considered when investigating the association between proteinuria and mortality. The HR for the total population remained unchanged after full adjustments, including adjustment for weight and BMI in the subgroup analysis. Moreover, results of subgroup analysis suggest that mortality in the obese group decreased by >40% compared to that in the non-obese group with ≥1+ proteinuria (Table 4A). The decreased mortality risk observed in overweight/obese subjects, known as the “obesity paradox,” could not be confirmed because of sample heterogeneity.[30] However, non-obese individuals with proteinuria should be stratified as a group with a higher risk of mortality than their obese counterparts.

Proteinuria is observed to be a better predictor of mortality compared to several established conventional cardiovascular risk factors. A previous study has demonstrated that the risk of future cardiovascular events such as stroke, myocardial infarction, and cardiovascular death was higher in patients with microalbuminuria than in those with peripheral artery disease or diabetes.[4] Current guidelines recommend proteinuria screening among high-risk populations such as elderly patients and those diagnosed with hypertension or diabetes.[31–36] However, our subgroup analysis indicates that all-cause mortality in individuals with ≥1+ proteinuria but without metabolic diseases (HR 1.513, 95% CI 1.468–1.559) such as hypertension, diabetes, or dyslipidemia was increased by >15% compared to that in individuals with negative or trace proteinuria and metabolic diseases in a fully adjusted regression model (HR 1.352, 95% CI 1.345–1.359) (Table 4B). This result corroborates the findings of previous studies and a feasibility study needs to be performed in non-risk groups to confirm whether dipstick screening is cost-effective when followed by administration of proteinuria-lowering medication in laboratory-confirmed cases.[37,38]

Previous studies have shown that trace proteinuria detected using a urine dipstick is a powerful predictor of mortality risk.[5] In a pooled meta-analysis that included 1.1 million individuals with a normal glomerular filtration rate, those with trace proteinuria showed an HR of 1.44 for all-cause mortality.[5] Our results showed that trace proteinuria showed an HR of 1.216 (95% CI 1.196–1.235) for all-cause mortality using a fully-adjusted regression model (Table 2), despite the concern about the attenuation of the mortality risk owing to the semi-quantitative nature of the test. Mild proteinuria is a treatable condition and timely and appropriate intervention can halt its progression.[39] Clinicians should be mindful of this fact and attempt to identify patients with even low quantities of proteinuria in the clinical setting. Increasing physical activity, reducing body weight, and appropriately treating hypertension or diabetes, as advocated for CKD,[39] would be an effective and valuable management strategy for patients presenting with trace proteinuria.

Limitations of our study: 1) Our results must be interpreted considering the variability in the manufacture and subsequent varieties of urine dipsticks, visual reading of dipstick strips in practice, role of exercise and infection, and intake of proteinuria-lowering medicines, and factors that could lead to exposure misclassification. 2) Although most centers that perform a general health examination in Korea use the Jaffe method for quantitative estimation of creatinine, reference ranges used might often vary between centers or laboratories.[40] Therefore, although PCR or ACR could be useful for confirmation of the results of our study, interpretation of results obtained from these tests was difficult. 3) Due to the nature of claims data, disease codes might not accurately represent the participants’ specific disease status, and drug prescriptions do not guarantee compliance; therefore, errors in the classification of comorbidities could be expected. 4) Study participants were Korean adults, and racial differences could influence the effect of proteinuria on mortality, and also lead to differences in the progression of renal disease associated with underlying conditions such as hypertension, diabetes, and proteinuria.[41] 5) Although hematuria with or without proteinuria has been suggested as an independent risk factor for the progress of CKD,[42,43] high false negative cases of dipstick hematuria could lead to another classification bias among study population.[44] Therefore, we excluded the variable of dipstick hematuria in current study.

## Conclusions

In conclusion, our study demonstrated that dipstick proteinuria was strongly associated with all-cause mortality. This finding provides a quantitative basis for the use of dipstick urinalysis in a clinical setting for risk assessment of all-cause mortality.
